# Facilitating the implementation of clinical technology in healthcare: what role does a national agency play?

**DOI:** 10.1186/s12913-018-3176-9

**Published:** 2018-05-10

**Authors:** Gill Harvey, Sue Llewellyn, Greg Maniatopoulos, Alan Boyd, Rob Procter

**Affiliations:** 1 Adelaide Nursing School, University of Adelaide, Adelaide, SA 5005 Australia; 2 Alliance Manchester Business School, University of Manchester, Booth Street East, Manchester, M13 9SS UK; 3 Institute of Health & Society, Newcastle University, Richardson Road, Newcastle upon Tyne, NE2 4AX UK; 4 Department of Computer Science, University of Warwick, Warwick, CV4 7AL UK

**Keywords:** Implementation, Technology, Facilitation, Insulin pump therapy, PARIHS, i-PARIHS

## Abstract

**Background:**

Accelerating the implementation of new technology in healthcare is typically complex and multi-faceted. One strategy is to charge a national agency with the responsibility for facilitating implementation. This study examines the role of such an agency in the English National Health Service. In particular, it compares two different facilitation strategies employed by the agency to support the implementation of insulin pump therapy.

**Methods:**

The research involved an empirical case study of four healthcare organisations receiving different levels of facilitation from the national agency: two received active hands-on facilitation; one was the intended recipient of a more passive, web-based facilitation strategy; the other implemented the technology without any external facilitation. The primary method of data collection was semi-structured qualitative interviews with key individuals involved in implementation. The integrated-PARIHS framework was applied as a conceptual lens to analyse the data.

**Results:**

The two sites that received active facilitation from an Implementation Manager in the national agency made positive progress in implementing the technology. In both sites there was a high level of initial receptiveness to implementation. This was similar to a site that had successfully introduced insulin pump therapy without facilitation support from the national agency. By contrast, a site that did not have direct contact with the national agency made little progress with implementation, despite the availability of a web-based implementation resource. Clinicians expressed differences of opinion around the value and effectiveness of the technology and contextual barriers related to funding for implementation persisted. The national agency’s intended roll out strategy using passive web-based facilitation appeared to have little impact.

**Conclusions:**

When favourable conditions exist, in terms of agreement around the value of the technology, clinician receptiveness and motivation to change, active facilitation via an external agency can help to structure the implementation process and address contextual barriers. Passive facilitation using web-based implementation resources appears less effective. Moving from initial implementation to wider scale-up presents challenges and is an issue that warrants further attention.

**Electronic supplementary material:**

The online version of this article (10.1186/s12913-018-3176-9) contains supplementary material, which is available to authorized users.

## Background

Accelerating the implementation of innovative technology in healthcare is an international policy concern [1]. In the United Kingdom (UK) – the setting for the study reported in this paper - the Department of Health published a policy to promote innovation in the National Health Service (NHS) in 2011 [2]. The policy set out a strategy for realising the potential of innovation to improve both quality and productivity; however, a subsequent evaluation indicated that progress towards achieving the strategic objectives has been somewhat patchy and slow [3]. This echoes the experience of implementing other changes in clinical practice. Empirical studies on knowledge translation, service improvement and organisational change consistently demonstrate that implementation is complex [4–7]. This has led to the development of implementation theories and frameworks that move beyond the notion of a linear pipeline [8] and attempt to represent the interactive, context-specific processes involved. Examples include Normalisation Process Theory [9], the Consolidated Framework for Implementation Research [10], Practice-based Theories [11] and the Promoting Action on Research Implementation in Health Services Framework (PARIHS) [4, 12].

In this paper, our aim is to examine the facilitation role of a national agency (the National Technology Adoption Centre or NTAC) in England charged with accelerating technology uptake in the NHS. We focus our analysis on the technology of insulin pump therapy (IPT) and study four organisations that received variable types and levels of facilitation support from NTAC. To frame the analysis, we apply the integrated-PARIHS framework [13] as a theoretical lens to compare the different facilitation approaches and evaluate factors that influenced implementation. Specifically, the paper aims to address the following research questions:

1. Why are some organisations more successful than others in implementing new technology? What factors influence success?
2. How does a national agency providing external facilitation support implementation?
3. How do active and passive facilitation strategies compare in terms of influencing implementation?

### Theoretical lens for analysis: The integrated-PARIHS framework

PARIHS (Promoting Action on Research Implementation in Health Services) was one of the first frameworks to explicitly recognise the complex, multi-faceted nature of implementing changes in practice. First developed in the late 1990’s [4], the framework has undergone 15 years of evaluation and refinement [12, 14, 15], resulting in a recently revised version known as the integrated-PARIHS (i-PARIHS) framework [13]. The basic proposition of i-PARIHS is that successful implementation results from the facilitation of an innovation with the intended recipients of implementation in their local, organisational and health system contexts. The key dimensions of i-PARIHS are summarised in Table 1.

**Table 1:** The i-PARIHS framework [13]

Successful Implementation = Facilitation (Innovation, Recipients, Context)	
Construct	Key elements
Innovation	Underlying knowledge sources (including research evidence, clinical and patient experience) Clarity Degree of fit with existing practice (compatibility vs contestability) Degree of novelty Relative advantage Trialability
Recipients	Motivation and readiness to change Values and beliefs Clinical consensus Local opinion leaders Skills and knowledge Time and resources Collaboration and team work Power and authority
Context	Leadership support Culture Past experience of innovation and change Structure, systems and processes Organisational priorities Policy drivers Incentives and mandates Inter-organisational networks and relationships
Facilitation	Network of facilitator roles (expert, experienced and novice)
	Facilitation processes to enable implementation:
	- Project management - Quality improvement - Team building and group process skills - Influencing and negotiating - Embedding and sustaining change

Facilitation represents the active ingredient of implementation. It comprises both facilitator roles and facilitation processes that may be internal or external to the implementation setting. Facilitation is concerned with enabling others (for example, by building knowledge, skills, relationships and confidence to enact change) as opposed to directing, telling, persuading or coercing people to change. This requires a flexible and iterative approach to assess and respond to barriers and enablers of implementation within the immediate and wider environment. We use i-PARIHS as a theoretical framework to examine the different facilitation strategies employed by NTAC, including how they addressed factors relating to the innovation, the recipients, and the local and wider context.

#### The technology of insulin pump therapy (IPT)

IPT is used in the treatment of Type 1 diabetes mellitus, a condition where individuals require lifelong treatment with insulin. This can be administered in one of two ways: multiple daily injections or a continuous subcutaneous infusion delivered by an insulin pump. IPT was the subject of a technology appraisal by the National Institute for Health and Care Excellence (NICE), which is independent from but accountable to the Department of Health in England. NICE guidance recommended IPT as a clinically and cost effective treatment option for adults and children with type 1 diabetes, where multiple daily injections had failed, and for children under 12 if multiple daily injections were not deemed practical or appropriate [16]. NICE also produced a Commissioning Guide to help health service commissioners plan and deliver services in line with the guidance [17]. This suggested uptake of IPT for Type 1 diabetes should be 12% of adults and 33% for children younger than 12 years old. Existing data suggested IPT adoption in the UK was much lower: around 1% in 2007 [18] and 3.7% in 2010 [19], in contrast to around 10% in other European countries [18]. It was this low uptake of IPT that prompted its inclusion in NTAC’s work programme.

#### The NHS Technology Adoption Centre (NTAC)

NTAC was established in 2007 as a small organisation of around seven staff, with a specific remit to tackle the under-utilisation of clinical technologies within the NHS in England. In 2013, after our study had finished, NTAC’s role and function were transferred to NICE.

The NTAC approach involved identifying a priority list of clinical technologies and then inviting NHS provider organisations (known as Trusts) to self-nominate as ‘implementation sites’ for a particular technology. From sites that applied, three or four were purposively selected and NTAC worked directly with them to facilitate implementation. A designated NTAC Implementation Manager acted as a facilitator to identify and engage with relevant stakeholder groups, within and outside of the Trust. NTAC also selected mentor sites – Trusts already implementing the technology without NTAC facilitation – to provide additional guidance and support to implementation sites. Learning from implementation and mentor sites was collated into an online toolkit, known as a ‘How-to-Why-to’ guide (HTWT), which included resources such as a template for developing a business case and a costing model for the technology [20]. The HTWT guide could be viewed as a passive facilitation strategy that was intended to enable other NHS organisations to implement the technology without direct, active facilitation support from NTAC. In this way, the aim was to achieve wider spread and scale-up of technology implementation.

## Methods

### Study design

The methodological approach was an empirical case study of four organisations with an interest in implementing IPT. This was one of three NTAC supported technologies studied as part of a larger research project to explore barriers to technology adoption in the NHS. The wider study has been reported elsewhere [1, 11, 21]. We selected four IPT sites, representing different levels of involvement with NTAC and/or different types of facilitation. Two sites (named Implementation Sites 1 and 2) received direct, active facilitation from an NTAC Implementation Manager; one site was a designated mentor (named Mentor Site) and had previously implemented IPT without external facilitation support; the fourth was a Trust that had applied to be an implementation site but was not selected (named non-NTAC site). This site was keen to implement IPT and, as such, was typical of the organisations that NTAC was targeting with passive facilitation, via the HTWT guide. Table 2 provides background descriptions of the 4 sites.

**Table 2:** Description of the case study sites

Relationship with NTAC	Description of case study site
Implementation site (1) (Direct relationship, active facilitation)	NHS Trust providing specialist children’s services, commissioned from a wide range of Primary Care Trusts (PCTs, 17 in total); differing arrangements in place in relation to funding and contractual agreements. Pressure to provide IPT had particularly been driven from the patient population (children and parents); as a consequence the diabetic team were keen to become more skilled and up to date in terms of providing pump services.
Implementation site (2) (Direct relationship, active facilitation)	NHS Trust providing acute services; formed from the previous merger of two Trusts. Diabetic services provided at two separate sites, one with a history of using IPT and the other less receptive to the new technology. No formalised processes or systems in place for managing the introduction of IPT at an individual patient or service level prior to involvement with NTAC. Commissioner (PCT) instrumental in applying for NTAC project.
Mentor site (Direct relationship, no facilitation)	NHS Trust providing an integrated hospital, community and primary care diabetic service. Originally applied to be part of the NTAC implementation project, but already well-advanced in terms of the implementation of IPT; as a consequence the organisation was invited to act as a mentor site for IPT. The Trust had an internal manager with responsibility for commissioning, who facilitated the relationship with the PCT to develop and negotiate contracts, an arrangement that worked particularly well in the introduction of IPT.
Non-NTAC site (No direct relationship, passive facilitation)	Specialist Diabetes Centre, hosted by an NHS Trust, having recently moved from a primary to secondary care setting, as a result of the changes to commissioning in the NHS. On account of its specialist status, the Centre dealt with a large number of commissioners (PCTs) across a wide geographical area. The introduction of IPT was led by a clinical academic, who had submitted an application to become an NTAC implementation site. This application was unsuccessful; however, the consultant and some of his colleagues had continued to try and develop their pump service without input from NTAC.

### Data collection

Data collection took place from July 2010 to July 2012 primarily using semi-structured, face-to-face interviews. A total of 23 interviews (mostly individual, one group interview) were conducted with key individuals involved in implementing IPT, including physicians, nurse specialists, commissioners, procurement and business managers. Three NTAC staff were interviewed: two Chief Executives (one present at the start of the study and a subsequent successor) and the Implementation Manager for IPT. Interviews were around 60 min long and were digitally recorded and transcribed for analysis. Table 3 summarises the interview sample by site and role.

**Table 3:** Interview sample by site and role

Site	Role	Number of interviewees
Implementation site 1	Diabetic Consultant	1
	Diabetic Specialist Nurse	2
	Contracts Manager	1
	Finance Manager	1
Implementation site 2	Diabetic Consultant	1
	Diabetic Specialist Nurse	2
	Project Manager (Dietitian)	1
	Commissioner	1
	Procurement Manager	1
	Finance Manager	1
Mentor site	Diabetic Consultant	1
	Diabetic Specialist Nurse	1
	Commissioning Manager	1
	General (Service) Manager	1
Non-NTAC site	Diabetic Consultant	4
	Diabetic Specialist Nurse	1
	Specialist Medical Trainee	1
	Clinical Manager	1
NTAC	Chief Executive	2
	Implementation Manager	1
TOTAL		26

Additional file 1 details the interview guide used within the overall research project. Supplementary data included background documentation, such as NTAC’s selection of the IPT implementation sites and notes of relevant meetings. To study the HTWT guides, we filmed five clinical staff and one patient advocate using the IPT guide in the presence of a research team member, using a ‘think-aloud’ method [22]. Participants were asked to articulate their thoughts on the content and ease of use as they navigated the HTWT guide in real time. The researcher asked reflective questions as appropriate. Finally, to gauge IPT uptake and awareness of NTAC and the HTWT guides, we undertook a short survey of clinicians who were members of a diabetes network with a special interest in IPT between May–July 2012 (Additional file 2). Table 4 summarises the data collection strategies used to address the research questions.

**Table 4:** Data collection strategies adopted to address the research questions

Research Question	Data source			
	Interviews	Documentation	Video- recordings	Survey data
1. Why are some organisations more successful than others in implementing new technology? What factors influence success?	✓			
2. How does a national agency providing external facilitation support implementation?	✓	✓		
3. How do active and passive facilitation strategies compare in terms of influencing implementation?	✓	✓	✓	✓

### Data analysis

Interviews were transcribed in full and, along with the documentary evidence, were analysed inductively using thematic analysis [23]. Core categories and themes were identified for each participant and then compared within and across the four cases. One member of the research team (GH) undertook the initial analysis. The wider project team then reviewed emerging categories and themes. Videos of participants using the HTWT guides comprised the computer display as seen by the user and a simultaneous shot of the user. A member of the research team (AB) analysed each of the videos, noting how the user navigated the HTWT guide and their verbal and non-verbal responses. Observations across the six videos were collated to produce key themes. We derived descriptive statistics from the diabetic network survey which had a response rate of 28% (91 responses from a sample of 320).

## Results

We present our analysis through the i-PARIHS core constructs: ‘Success’ of implementation; Innovation (IPT)-related factors; Recipient-related factors; Context-related factors; Facilitation-related factors.

### ‘Success’ of implementation

Of the two sites receiving direct, active facilitation from NTAC, Implementation Site 1 increased the numbers of patients using IPT during the timescale of the project from around 9 to 53. In Implementation Site 2 the numbers using IPT stayed around 100, but this figure masked the fact that some patients were newly started on pumps and others were taken off because they did not meet the NICE criteria for IPT.

The qualitative data indicate that the two implementation sites engaged actively in the implementation project and made progress in developing their pump service. Interviewees spoke positively about the support they received from the NTAC Implementation Manager, who worked across both sites, and of their overall experience of the project. The NTAC project was seen to have provided a catalyst and a structure to move forward on an issue that they were committed to improving. Prior to their involvement with NTAC, the decision to place patients on pump therapy had been mostly consultant-led, with funding organised on an individual patient level. Working with NTAC had provided a clear project management approach to address issues such as developing an agreed care pathway for pump patients, joint working with commissioners to address funding arrangements, establishing a clear procurement process for pumps, developing the necessary nursing knowledge and skills to manage patients on pumps, undertaking regular audits and introducing multi-disciplinary pump clinics.

Both implementation sites and the mentor site were involved in the development of the HTWT guides; however, the extent to which other Trusts asked for information or advice following publication of the guide was limited. It was viewed unlikely that the guide could substitute for the hands-on project management support that NTAC provided.

*I’d like to be able to show you some examples of where this website has been used and someone’s done what we have done, but my worry is that it’s quite hard without that push from NTAC centrally.* (Consultant, Implementation Site 1).

This view was reinforced in the data from the non-NTAC site where the pump service was similar to that in the two implementation sites prior to their involvement with NTAC. The service was championed by an individual consultant but had no secure funding model in place and lacked a clear infrastructure (such as specialist nurses) to develop it further. The lead consultant was sceptical about how the HTWT guide would help to address these issues:

*I have to admit that I haven’t really looked at it and I think that’s because I’ve got the impression, from other pump professionals …. that, you know, it’s not going to change my world for pump patients … no-one’s kind of saying, okay you download that document, take it to your Trust, you’ll be sorted.* (Consultant 1, non-NTAC site)

The survey findings raise further questions about the impact of the HTWT guide. 54% (*n* = 49) of the survey respondents were aware of NTAC; of these, 63% (*n* = 31) were aware of the HTWT guide on IPT. Only 13% (*n* = 6) had used the guide to develop a business case for implementing IPT and even fewer (8%, *n* = 4) had used it to contact other organisations for information. Video data from real-time users of the HTWT guide indicated difficulties in navigating the online resource, partly due to the way the content was organised and also because of the slowness of the website. Some users felt that the content portrayed the process of implementation as too complex, time-consuming and technical.

In summary, sites that received direct facilitation support from NTAC reported the most positive outcomes in terms of establishing or expanding their provision of IPT, including setting up the necessary infrastructure and funding arrangements. The same level of progress was not observed in the non-NTAC site and the data indicate limited impact of the HTWT guides beyond awareness of their existence.

### IPT-related factors

Some clinical staff expressed a view that issues relating to the acceptability of pumps to patients could contribute to a lack of uptake. These included concerns about how the pump might fit with a patient’s lifestyle or how they felt about being permanently attached to a pump; for others it was the requirements that went alongside using a pump, for example, the need for active self-management and regular blood glucose monitoring.

*There are a significant number who think about it and then just don’t want even to take anything any further. …… Having seen one, talked about it in more detail, quite a number of them have come to the conclusion that it’s not for them ....* (Consultant 2, Non-NTAC site)

For others the pump was seen to provide greater freedom to manage their diabetes, despite initial concerns about the pump itself:

*..... I think the patient experience is honestly amazingly positive. Even people who have apprehensions about going on the pump … generally speaking a few months into pump they would not go back to injections.* (Specialist Medical Trainee, Non-NTAC site)

Despite evidence from clinical trials and a national technology appraisal of IPT, some clinicians doubted whether there was sufficient evidence to support the use of IPT.

*I’m not even a hundred per cent sure about the proven clinical benefits. If you … do the proper randomised controlled trial, pump* versus *intensive therapy with an equivalent amount of input from health care professionals, you don’t get a great difference in HbA1c* [a measure of average blood sugar levels] *or in anything else. You might get a difference in patient satisfaction in favour of a pump, but that’s in folk who want to go onto the pump. So I think sometimes the benefits of pumps are a bit overstated.* (Consultant 2, Non-NTAC Site)

This was in contrast to the other sites where IPT was widely regarded as a ‘proven technology’.

*I suppose, on the surface, it looks expensive but there’s then the evidence.... that’s why NTAC have taken it on ..... because the improvement of the blood result [and] …. in preventing complications ...[it] is a proven technology for improving progress with [diabetic] complications.* (Diabetic Specialist Nurse 1, Implementation Site 2).

A particular issue was raised about the wording of the NICE guidance, which indicated IPT as the recommended treatment option when multiple daily injections had failed (in adults) or were seen as impractical or inappropriate (in children). This created an interpretation of IPT as a ‘treatment of failure’, rather than a clinically effective option that should be available to all.

*…. NICE guidance is geared to pump use being used as a treatment of failure. So if you fail with your multiple daily injections,* i.e. *you’ve still got poor control, or you’ve still got low blood glucose. It’s a negative process ... … Why is pump therapy used as the treatment of failure, if it’s supposedly the best treatment?* (Specialist Medical Trainee, Non-NTAC site)

### Recipient-related factors

As already noted, considerable divergence of opinion was apparent. This included differing views around who was responsible for driving increased use of IPT. Some clinical staff felt that patients were responsible for the growth of the technology:

*We are very good at being ahead of our patients in what they know about either their condition or their treatment plan or their medications. But with insulin pumps, there was a feeling that we were only just one step ahead of our patients …… the patients were asking for them and we were saying, “Oh, hang on a minute. We’re not so sure that we have the skills and knowledge to facilitate this for you.”* (Diabetic Specialist Nurse 1, Implementation Site 1)

Other clinicians did not perceive a significant patient-led demand for pumps:

*One of the things that I have noticed when I’ve been to meetings and so on elsewhere that some of the people say patients ask for pump therapy. It doesn’t happen in my experience very much. .... it tends to be us that says, “well actually I think you might benefit, would you consider a pump?”* (Consultant, Implementation Site 1)

Clinician related factors appeared particularly influential in the decision to use IPT. The motivation of individual consultants was especially important.

*.... we have always been a forward looking trust I think, although we are only a district general hospital I do think that we have a philosophy ….. that our patients should not miss out on any treatment that could be of benefit to them* (Consultant, Implementation Site 1).

The motivational issue also worked in reverse and created a barrier to implementation if individual clinicians were wary or resistant to use the technology. In some cases, this related to a negative experience with a pump:

*I’m sceptical because I’ve seen the good things and I’ve also seen the bad things about pumps. Like the girl we admitted last weekend …. who nearly died … we hadn’t put her on a pump, she was put on a pump elsewhere and she didn’t self-manage properly.* (Consultant 2, Non-NTAC site)

In other cases, resistance was tied up with local politics and personality differences:

*…. one of the … internal barriers to implementation … one individual in particular who was asked to be involved …. but if it isn’t his project he doesn’t want to know and he has done quite a few things to try and sabotage the* [project]*.* (Project Manager, Implementation Site 2)

Alongside the motivational (‘want to’) factors, issues relating to capability and capacity (‘can do’ factors) to provide an IPT service were also identified. These included the need for staff and patient education:

*When a patient goes on a pump, initially, it is...very time-consuming, the patient needs lots of education .... the thing about the pump is, whether it works or not, depends on patients’ self-management.* (Diabetic Specialist Nurse 2, Implementation Site 1)

Some clinicians expressed a view that the additional input required to care for pump patients could actually disadvantage non-pump patients:

*…. in [this organisation], we expend a great deal of health care time with the small number of people who are on pumps and that is to the detriment of people who are not on pumps. We have a limited number of physicians, nurses, dieticians and if they’re working with pumps, it means that they’re not working with our other non-pump population.* (Consultant 2, Non-NTAC Site)

### Context-related factors

Beyond the patient and clinician level, there were factors relating to the organisation and delivery of healthcare that impacted on IPT provision. These included politics and culture at a local level, alongside organisational and system level issues related to funding and commissioning new technologies. Introducing IPT required a significant upfront investment to purchase pumps, alongside the necessary infrastructure to educate and support staff and patients, including the appointment of specialist pump trained nurses. How this funding was obtained varied considerably. Historically, some clinicians had funded IPT through their own research budgets. However, the publication of the NICE guidance had placed a requirement on commissioners of health services to fund IPT, which was seen to be an important driver for implementation.

*PCTs* [Primary Care Trusts, the organisations responsible for commissioning healthcare in the English NHS at the time of the study] *…. have to be seen to be following NICE guidance, so I know it’s a time of austerity and the PCTs have other priorities, but a trust should always play the NICE guidance card and say, look you know, that’s why we’ve got NICE guidance because it’s the best outcomes for this group of patients.* (Diabetic Specialist Nurse, Mentor Site)

As indicated in Table 2, each of the sites had differing arrangements with their commissioners and this influenced the ease with which they could implement IPT. The closer and less complicated the relationship, the smoother the process appeared to be:

*… it’s been driven primarily by the PCT because once I became involved, I did actually start trying to structure it and say, you know, let’s look at it in a more comprehensive way; rather than just clinicians thinking this is a great thing to do, without any thought about how we’re going to pay for it.* (Commissioner, Implementation Site 2)

This was in contrast to Implementation Site 1 that worked with many different commissioning organisations, creating an added complication because “*they all have a different way of working*” (Diabetic Specialist Nurse 2, Implementation Site 1). NTAC placed a lot of emphasis on helping organisations navigate issues related to funding IPT, through providing help with developing business cases and producing costing models. However, interviewees raised concerns that the costs related to establishing an IPT service, namely a nurse specialist and patient education, typically fell outside PCT funding arrangements and had to be directly absorbed by the provider organisation.

### Facilitation-related factors

Here we focus on the two different strategies (either active or passive) that NTAC employed to facilitate implementation. The initial active facilitation approach involved an NTAC Implementation Manager working as an external facilitator and using project management methods to structure the implementation process. This was followed by a more passive facilitation strategy that relied on the HTWT guide, an interactive web-based resource, without direct facilitator input. For the two implementation sites, the NTAC facilitator played a key role. Whilst the sites were motivated to implement IPT, the external facilitation provided by NTAC acted as a catalyst and helped to guide the implementation process through providing a formal project management structure and developing the systems that were needed to support the use of the technology.

*They [NTAC] were phenomenally helpful. We knew where we wanted to be, but weren’t sure of the map to use to get us there..... NTAC were really good in helping us to get the people in the room who needed to be in the room, to have the right conversations. Project management – I think that’s what we really lack and what they did really well.* (Diabetic Specialist Nurse 1, Implementation Site 1)

This strong project management focus was in keeping with how NTAC perceived their role in implementation, although interestingly there was no agreed framework to guide their approach.

*There was no model. There was no laid out plan of how to do it ... [the chief executive] wanted people who could come in, who were experienced, who had enough seniority to push this change forward. To be in that environment, you needed to have some initiative of your own ... [the chief executive] said to me, ‘I don’t care how you do it ... Here’s your budget. Now go away’ ... I had a blank piece of paper.* (NTAC Implementation Manager)

Whilst NTAC provided a welcome project management approach, their involvement was perceived to be at the planning stage of the implementation process. Implementation Site 2 had an internal project manager who was responsible for the ongoing implementation; in Implementation Site 1 it was less clear where this responsibility lay. The active process of facilitation, with an individual in a designated facilitator role, was in sharp contrast to the planned process for rolling out implementation of IPT through the HTWT guide on an interactive website. Respondents expressed considerable doubt over whether this more passive approach could substitute for the hands-on facilitation provided by the NTAC Implementation Manager.

## Discussion

From the findings it is apparent that evidence perceived to be ‘strong’ from a traditional healthcare evaluation perspective (i.e. confirmed by multiple randomised controlled trials, systematic reviews and technology appraisal) could be subject to different interpretations at a patient and clinical level. This was clearly so in relation to IPT despite the NICE guidance. There was a juxtaposition of evidence of effectiveness with questions of acceptability or appropriateness, for example, whether patients wanted to wear a pump or whether they met the criteria for self-management. In other situations, the evidence of effectiveness was itself the focus of debate and individual clinician experience could cast doubt on its robustness or relevance, as was the case where a medical consultant had witnessed a critical incident of a patient using IPT. This reinforces other studies that demonstrate the contestability of research evidence [5, 24–26]. It also illustrates the complex inter-relationship between the innovation and the intended targets of innovation, relationships that directly influence and shape the innovation journey [27, 28]. Additionally, it demonstrates the mediating role of context, not just at the immediate level of implementation but at the policy, systems and organisational level, where the complexities associated with the business of healthcare delivery (for example, funding, commissioning, procurement) are important determinants of whether and how easily a new technology can be implemented [11].

What do the findings tell us about the role of a national agency such as NTAC with a remit to function as a facilitator of implementation? They suggest that NTAC did play a central role in facilitating the implementation of IPT in the two sites they worked with. At both sites a set of circumstances were observed that created a high level of receptiveness to IPT. The evidence underpinning the technology was well accepted by clinicians, managers and commissioners and strengthened by formal endorsement through NICE. In Implementation Site 1, this was further enhanced by the patient/parent push to have access to the technology. Thus there was a clear alignment of stakeholders around the innovation, in terms of its relevance, acceptability and effectiveness. This created a strong motivation to introduce IPT, which is recognised as an important element of readiness to change [29]. However, the capacity to change was less apparent as the organisations were generally uncertain about how to approach implementation. This is where the active facilitation provided by NTAC was beneficial, with the NTAC Implementation Manager helping to bring key stakeholders together, providing a structured project management approach and addressing system issues related to funding, commissioning and procurement of the technology.

In contrast the non-NTAC site demonstrated less agreement around the innovation, in terms of whether it was safe or had a strong enough evidence base to support its use. One clinician was extremely keen to introduce pump therapy but did not have the full support of colleagues, management or the wider multi-disciplinary diabetes team. Arrangements for funding IPT were unclear and progress with implementing the technology was limited. Whether the NTAC approach would have worked as easily or smoothly in such a setting is debatable, as here it was not simply a case of introducing a project management structure and process. Knowledge could be seen to be ‘at stake’ [30] as professionals within the clinical team held opposing views and interpreted the evidence for IPT in different ways. Consequently, more significant boundaries to implementation existed, likely requiring a more intensive and tailored approach to facilitation, with greater attention to negotiating the differences of opinion [30, 31].

The mentor site did not work with NTAC or an external facilitator to introduce IPT. However, there was clear evidence of facilitating roles and processes within the organisation that were key to enabling implementation. The organisation had well established networks and relationships across acute and primary care and could be described as an early adopter [27] as they had introduced IPT sometime before NICE guidance was issued. This integrated way of working was supported by having a manager based in the acute service with an explicit responsibility for working with primary care commissioners to agree contracts. This manager effectively functioned as an internal facilitator of implementation and the financial and system level barriers that were problematic in other organisations did not present a major obstacle. This highlights the potential importance of an internal facilitator to embed and sustain implementation, a role that the designated project manager in Implementation Site 2 appeared to fulfil.

The i-PARIHS framework suggests that the dynamic relationships between the innovation, intended recipients and the context are best handled through the active medium of facilitation, defined in terms of facilitator roles and facilitation processes. The findings of our study largely support this proposition, highlighting the importance of active facilitation via an individual functioning in a facilitator role. NTAC’s strategy for wider roll-out of IPT which involved moving from direct support by a designated facilitator to an online resource (in other words, from active to passive facilitation) met with limited success, suggesting that some form of human agency facilitating implementation is important. However, exactly what nature, form and function the facilitation process should take merits further exploration. For example, is a project management approach to facilitation (as adopted by NTAC) particularly suited to organisations with a high level of initial readiness and receptiveness to innovation? And is this sufficiently flexible where there are less receptive conditions? Alternatively, when there is an extremely favourable set of circumstances (as in the case of the mentor site) is an environment where internal managers function in a facilitative way sufficient? These are all interesting and important areas for further investigation, particularly in the context of debates about whether we are over-engineering implementation strategies when simple and cheaper alternatives would suffice [31, 32]. Further questions relate to how organisations with a national remit for implementation can best fulfil their role if facilitation via human agency is required. From a resource perspective, it may be more appropriate for agencies such as NTAC to work through local networks (such as the Diabetic Network involved in the survey) to develop and cascade the facilitation skills needed to implement, sustain and spread new technology, rather than providing direct facilitator support to a small number of organisations [33].

## Conclusions

This paper has analysed an empirical case study of four organisations attempting to implement IPT to illustrate the interplay of factors that influence the implementation of clinical technology in healthcare and the role of a national agency in facilitating the process. Through the conceptual lens of the i-PARIHS framework, the findings indicate that when favourable conditions for implementation exist, in terms of agreement around the innovation to be implemented and clinical receptiveness and motivation to change, a national agency can employ a combination of an external facilitator and project management methods to initiate implementation and address contextual barriers relating to funding and commissioning new technology. The extent to which such an approach would achieve similar results in a less receptive environment is unclear. There is also the important issue of how a national agency can best facilitate the spread of innovation since moving from active to more passive methods (in this case, direct support from Implementation Managers to an online resource) appeared to have little impact.

We acknowledge that a potential limitation is our focus on the study of a single technology, which may limit the transferability of the findings to the wider challenge of technological innovation in healthcare. Equally we recognise that our findings reinforce similar issues identified in other studies of technology implementation and uptake in healthcare [34, 35]. We believe there is merit in applying implementation theories to inform this field of study, both prospectively and retrospectively. In particular, we would suggest further examination of the facilitation roles and processes needed to work with organisations that are reluctant or late adopters of new technology, including strategies that can be scaled up in both an effective and efficient way.

## Additional files

Additional file 1: Copy of interview guide used in the study. (DOCX 19 kb)

Additional file 2: Copy of survey questions. (DOCX 101 kb)

## Abbreviations

HTWT: How-To-Why-To
i-PARIHS: Integrated-Promoting Action on Research Implementation in Health Services framework
IPT: Insulin pump therapy
NHS: National Health Service
NICE: National Institute for Health and Care Excellence
NTAC: National Technology Adoption Centre
PCT: Primary Care Trust
