# Correction to: Facilitating the implementation of clinical technology in healthcare: what role does a national agency play?

**DOI:** 10.1186/s12913-018-3216-5

**Published:** 2018-06-11

**Authors:** Gill Harvey, Sue Llewellyn, Gregory Maniatopoulos, Alan Boyd, Rob Procter

**Affiliations:** 1 Adelaide Nursing School, University of Adelaide, Adelaide, SA 5005 Australia; 2 Alliance Manchester Business School, University of Manchester, Booth Street East, Manchester, M13 9SS UK; 3 Institute of Health & Society, Newcastle University, Richardson Road, Newcastle upon Tyne, NE2 4AX UK; 4 Department of Computer Science, University of Warwick, Warwick, CV4 7AL UK

## Correction

Upon publication of the original article [1], Gregory Maniatopoulos’ name was incorrectly given as ‘Greg Maniatopoulous’. This has now been corrected in the original article.
