# Supplementary material for: Facilitating the implementation of clinical technology in healthcare: what role does a national agency play?
Source: BMC Health Serv Res. 2018 May 10;18:347. doi: 10.1186/s12913-018-3176-9 (PMC5944036; doi:10.1186/s12913-018-3176-9)
Supplement: Supplementary file 1 — Copy of interview guide used in the study. (DOCX 19 kb) [file 12913_2018_3176_MOESM1_ESM.docx]

Llewellyn S, Procter R, Harvey G, Maniatopoulos G, Boyd A. Facilitating technology adoption in the NHS: negotiating the organisational and policy context - a qualitative study. Health Serv Deliv Res. 2014;2(23).

**Interview Guide: Some questions will be more relevant than others dependent upon the role of the participant so questions may be omitted or probed further as appropriate. Semi-structured format, all interviews in a face-to-face situation at the Trust. All answers to be taped and transcribed, if acceptable to participants.**

**General questions**

Q1. Please will you outline your role in working towards the successful adoption and implementation of (name of specific clinical technology).

Q2. What are the main clinical benefits of (name of specific clinical technology)?

Q3. What do you think are the main organizational barriers to adoption and implementation?

Prompt: raises costs; funding issues; organizational inertia; clinical resistance; managerial resistance, difficulty in forming the business case; commissioning issues

Q4. What do you think are the main policy barriers to adoption and implementation?

Prompt: PbR, organizational targets (eg waiting lists), competing policy objectives, rapid policy reform

Q5. Are there any unresolved clinical barriers to adoption and implementation?

Q6. Do you believe that adopting and implementing (name of specific clinical technology) is risky? If, yes, how is this risk perceived? And how is this risk alleviated?

**Building a support network**

Q7. Was there key individual who championed the adoption of (name of specific clinical technology)? If, yes, why was this person so crucial and would the technology have been adopted without them?

Q8. Who are the key individuals *in the Trust* whose support was required for successful adoption?

Q9. Who are the key individuals *outside of the Trust* whose support was required for successful adoption?

Q10. What were the main issues in building this network of support?

Q11. Has this network been sustained? If, yes, how? If, no, why?

Q12. Were there any individuals who were resistant to the adoption of (name of specific clinical technology)? If, yes, why were they resistant? And how was their cooperation secured?

Q13. Did successful adoption necessitate the creation of new organizational roles? If, yes, what were these roles? Did any of these roles cross boundaries? Prompt: between the Trust and commissioners; between the Trust and suppliers; across different specialisms within the Trust.

**Payment by Results (PbR) and funding issues**

Q14. Do you think that PbR created particular difficulties for the introduction of (name of specific clinical technology)? If, yes, what were these?

Q15. Is the Trust engaged in strategies to try to resolve these difficulties? If, yes, what are these strategies?

Q16. Do you believe that (name of specific clinical technology) will eventually raise productivity? If yes, why? If no, why not?

Q17. Did the introduction of HRG4 impact on (name of specific clinical technology)? If, yes, what was the impact?

Q18. Is the Trust engaged in any new costing initiatives as a result of the introduction of

(name of specific clinical technology)?

**User acceptability issues**

Q19. Were there clinical user acceptability issues in relation to (name of specific clinical technology)? If, yes, what were these and how were these overcome?

Q20. Were work processes disrupted due to the introduction of (name of specific clinical technology)? If yes, what form did this disruption take and how was it overcome?

Q21. Will the benefits of the introduction of (name of specific clinical technology) accrue solely within the Trust or will there be benefits outside? Prompt: Primary care; home care

**Patient acceptability issues**

Q22. Were there any patient acceptability issues in relation to (name of specific clinical technology)? If, yes, what were these and how were these overcome?

Q23. Does the effective use of (name of specific clinical technology) require patient/carer compliance/involvement/knowledge? If yes, what work with patients/carers was required to ensure the effective use of the technology?

Q24. Were patients involved in either the adoption or implementation processes? If yes, what form did this involvement take?

Q25. Has patient feedback on (name of specific clinical technology) been sought? If yes, are you aware of any issues that this feedback raised?

**Supplier/Industry issues**

Q26. Was the Trust encouraged by the producers of (name of specific clinical technology) to adopt? If yes how was this manifested?

Q27. Was the Trust well supported by the producers of (name of specific clinical technology)?

If yes, what form did this support take? If no, what problems did this create?

**Engagement with NTAC**

Q28. How has your engagement with NTAC assisted in the adoption and implementation of

(name of specific clinical technology)?

Q29. Do you believe than there would have been adoption without NTAC’s support?

Q30. Is your engagement with NTAC still on-going? If, yes, what form does this take?

Q31. Has NTAC produced a ‘How to, Why to’ Guide for (name of specific clinical technology)?

Q32. Will this How to, Why to guide will be of any relevance to you? Or do you think that your adoption process can now be considered complete?

Q33. Do you know if NTAC has produced any other ‘How to, Why to’ Guide for another technology which you are considering adopting? If yes, have you downloaded this guide? How has the guide helped you?

**Closing questions**

Q34. Do you believe that the difficulties the Trust experienced in adopting (name of specific clinical technology) are specific or would they apply to other technologies?

Q35. Do you think that best practice can be prescribed for technology adoption? If yes can you indicate what form this would take?

Q36. Reflecting on the adoption and implementation process for (name of specific clinical technology) if you were to undertake this process again what would you do differently and why?

**Thank you for agreeing to participate in this research**
