# Supplementary material for: Facilitating the implementation of clinical technology in healthcare: what role does a national agency play?
Source: BMC Health Serv Res. 2018 May 10;18:347. doi: 10.1186/s12913-018-3176-9 (PMC5944036; doi:10.1186/s12913-018-3176-9)
Supplement: Supplementary file 2 — Copy of survey questions. (DOCX 101 kb) [file 12913_2018_3176_MOESM2_ESM.docx]

Llewellyn S, Procter R, Harvey G, Maniatopoulos G, Boyd A. Facilitating technology adoption in the NHS: negotiating the organisational and policy context - a qualitative study. Health Serv Deliv Res. 2014;2(23).

Top of Form

*This survey explores issues relating to Continuous Subcutaneous Insulin Infusion (CSII). It contains 7 multiple choice questions and should take no more than 3 minutes to complete.*

Have you been involved, at your Trust, with increasing the number of patients with Type 1 diabetes who use CSII? .

- Yes
- No

If no, please go to end of survey

If yes, could you estimate the current percentage of patients, at your Trust, with Type 1 diabetes who are now using CSII?

- 0 - 5 %
- 5 - 10 %
- 10 - 15 %
- Over 15 %

Please could you now estimate the percentage of patients with Type 1 diabetes that were using CSII 3 years ago?

- 0 - 5 %
- 5 - 10 %
- 10 - 15 %
- Over 15 %

Are you aware of an organisation called the NHS Technology Adoption Centre (NTAC)?

- Yes
- No

If no, go to end of survey.

On their website, the NHS Technology Adoption Centre (NTAC) have a 'How-To, Why-To' (HTWT) guide for CSII adoption and implementation, are you aware of this guide?

.

- Yes
- No

If no, go to end of survey

If yes, have you used this guide for (tick all that apply)

- A business case to your Trust to develop your CSII service
- A business case to your commissioners to develop your CSII service
- An information resource about CSII
- To contact other Trusts with experience of developing their CSII service

On a scale of 1-5 how helpful did you find the NTAC HTWT CSII guide?

| 1 (Extremely unhelpful) | 2 (Somewhat unhelpful) | 3 (Neutral) | 4 (Somewhat helpful) | 5 (Extremely helpful) |
| --- | --- | --- | --- | --- |
|  |  |  |  |  |

Please use the box below for any further comments

*Thank you for your time.*

Survey Powered By [Qualtrics](http://www.qualtrics.com)

Bottom of Form
